# Mr. Mortimer's Report of the Yellow or Bulam Fever

**Published:** 1817-03

**Authors:** John Mortimer


### Mr. Mortimer's *Report of the Yellow or Bulam Fever.*

( Continued from our last Number, page 101.)

IT has been shewn in the history of the Regalia, that she continued perfectly healthy during the period of her stay on the African coast, until she received a quantity of green wood. We have it lamentably in proof, that very soon afterwards fever made its appearance, and that it continued with unabated malignity on her arrival at Barbadoes, where it was believed that she had imported in the persons of African recruits, a highly pestilential disease ; and, where, if quarantine laws had been in force, she would assuredly have been interdicted all communication with the shore, and consequently more lives would have been lost for want of such preventive and curative measures, as (when at length happily adopted) were found to be successful. We have it furthermore in evidence, that the process of fumigation is insufficient to destroy the noxious qualities of putrescent vapours ; and that it is only by a complete clearance of every thing offensive that we can reasonably hope for positive security.

That some matter highly inimical to health did exist in this ship, and that it was of the most powerful and malignant kind, has been evidenced by its speedy action on all such as were engaged from time to time to fill the places of the dead : and if farther proof were desired by the incredulous, it might be adduced in its fatal operation on the cook, a man upwards of fifty years old, and the only one of the whole crew who had, up to the period of clearing out the hold, been exempted from seizure.

This ship has been particularized, not only as being the most recent instance of the decided effect of miasmata on the human frame, but also because to the additional weight of Dr. Ferguson's recommendation is to be ascribed her prompt and effectual clearance.

Green wood stowed in contact with bilge water, has been stated as a probable cause of fever. This presumption at least is in its favour, that previous to its reception, health prevailed; very soon afterwards disease succeeded; and not until the full clearance of the vessel did it cease.

In its effect, the African partook quite as much as the European; and whatever discrepancy of opinion may arise as to the character of the fever affecting the former, of its existence and highly inflammatory nature, as evidenced by the blood drawn from the arm, there cannot be any doubt

Referring to Dr. Chisholm's description of the internal condition of the Hankey, and of the fever there prevailing, we are forcibly struck with the similarity of these two vessels. In each there would appear to have been a neglect of every proper regulation for a conservation of health; in each, a total apathy to the dangers often resulting from the want of cleanliness.

It deserves particular notice, that the arguments advanced in favour of the propagation of this disease from the Hankey to the people on shore are but few, and those by no means strongly supported; whilst the circumstance of repeated cases of its attacking persons who had several times visited the ship, accords with the experience of many who, although they admit the adequacy of miasma to produce fever in those who are long or often exposed to its influence, nevertheless deny to it any infectious property, in the full persuasion that to miasma and not any other peculiar character, might the fatality be ascribed, which occurred on board that particular ship.

In whatever points of view the consideration of the letter of the Honourable Board be taken up; whether in the discussion of the various and opposite theories advanced, respecting the seat and nature of fever, and to compare the conclusions resulting therefrom in relation to its treatment,—or in the support or refutation of the novel doctrine of the contagious properties of the one peculiar to warm climates, — the importance of the subject must be acknowledged by all: for to the diversified opinions still entertained, may we justly attribute the alarm which invariably attends the first appearance of an epidemic fever, leading, not unfrequently, to the failure of the most extensive and best planned expeditions.

It may be presumed that, under such an impression, his Majesty's government has interested itself in the circulation of Mr. Pym's publication, for the avowed purpose of exciting more general inquiry, and thereby drawing from extensive sources such communications as, it may be hoped, will materially assist an unbiassed judgement in determining the weight that should be allowed to the supposition of its contagious property; and also, which is of the greatest moment, to lay down some more general and defined mode of treatment than has hitherto obtained.

That the fevers of the Mediterranean, of Gibraltar, and of the West Indies are the same in kind, differing only in the degree of intensity, I fully believe: that the progress of all may be much controlled by the skill of the physician, successful practice has, I think, firmly established.

But when we are told by an Author, who, from his high official situation, has the power of enforcing his doctrines, that there is a yellow fever in which blood-letting is serviceable, and a "Bulam" in which it is inadmissible;—when, moreover, in the attentive perusal of his book, we discover the strong prejudice every where evinced by him *to its adoption*, it becomes every man who entertains a contrary opinion, however inferior his powers of language may be, to step forward in its defence.

In examining the bodies of those who have died of the yellow fever without having suffered much affection of the stomach, I have generally found the meninges of the brain highly *vascular*, and oftentimes the minute vessels of the viscus itself greatly distended with blood, its surface patched with lymph, and the adhesions remarkably firm. In other cases, where the stomach has been more particularly the seat of disease, the mottled appearance of its internal membrane, and the dissolved texture of the coats in general, have strikingly exemplified the celerity with which disorganization succeeds to high inflammatory action unsubdued by curative measures.

That such appearances as I have just described, accord with the observations made by those who have had similar opportunities, is well known.

The means by which we can most reasonably hope to prevent such a state are yet subjects of discussion and controversy; which, for the credit of medical science as well as for the sake of suffering humanity, it is most fervently to be wished, might be soon brought to a conclusion.

The primary indication is the removal of congestion, best evidenced by the subsidence of pain. This we cannot expect to accomplish while the lancet is so reluctantly and inefficiently employed, owing to the fear of debility, which neither the publication of Mr. Watts on Diabetes, nor the successful adoption of this remedy in Dropsy, as recorded by Dr. Blackall, has been able to remove. For the fulfilment of this indication, blood must be drawn in a full stream, (and it matters not from whence) not by a careful measurement, but till relief is obtained, or syncope induced.

Objections have been made to this energetic practice, from the insidious manner in which the disease sometimes steals on the patient. With symptoms more indicative of dyspepsia than of high febrile action, those most remarked being a pale moist tongue, dejected countenance, vertigo, a watery eye, and coldness of skin, an emetic has been administered as the appropriate remedy. A short time only however has elapsed, ere the true character of the disease has manifested itself, accompanied by irritability of stomach, if not induced, at least aggravated, by what was designed to afford relief.

This deceptive form of fever has been repeatedly observed by myself, as well as by my distinguished predecessor Dr. M'Arthur. In cases of this sort, instead of soliciting the inverted action of the stomach, I have lost no time in immersing the body in warm water, which, by exciting the languid circulation, and giving to the surface its natural warmth, has paved the way for the introduction of the lancet. And I may here observe, that until the system is thus prepared, the abstraction of blood is not only useless but even injurious.

In recommending bleeding, I would have it borne in recollection that it is not the only remedy that is required.

Dissection has seldom discovered in the liver or its ducts any peculiar appearances, nor are the contents of the gall bladder visibly altered; yet that the biliary secretion in the progress of yellow fever is always vitiated, is evident from the dark colour of the fæces; and though sometimes the lower belly is loose, there are retained in the cells of the colon a considerable quantity of scybala.

The importance of completely emptying the intestinal canal in the early stage, and of inducing a more healthy secretion of bile, must be manifest. For this purpose calomel joined with jalap is in general estimation, and should be repeated in small doses as long as the febrile heat continues. Clysters are of the greatest service throughout the disease; and where a more open state of the belly is not deemed necessary, the frequent injection of some warm fluid has a considerable effect in quieting the general irritability.

The most distressing accompaniament is vomiting; and having often experienced the happy effects produced by the disengagement of fixed air *in the stomach* from the union of carbonates of magnesia and ammonia, followed by a draught of lemon juice, I beg to recommend it to the attention of practitioners.

The shower bath and clysters, where pain continues, are excellent remedies. The former should be repeated as often as morbid heat returns, and the latter applied as large as possible, and so soon as the lancet and purgatives have been premised.

These are the means on which we may in general rely in the treatment of fever.

Much has been written on the virtues of mercury, and there are those who still insist on the pre-eminent rank to which it is entitled. Calomel, the preparation in general use, was exhibited by me whilst at Mariegalante, in doses from three to eight grains every third hour. Mercurial ointment was rubbed on the thighs and legs without restriction to quantity, the only limitation being with regard to the duration of each friction, as depending on the ability of the patient to bear it; but in few instances, and those only as a purge, did it evince any positive effect. The fever subsiding, salivation has come on; but however profuse, and consequently however protracted the recovery of the convalescent, it offered no additional security against future seizure.

From the high recommendation of Dr. Chisholm I began its use, in confident assurance of much success; but not only at Mariegalante but at all the public situations which I have subsequently held, the only effect I have known to result from calomel, during the continuance of high febrile action, was as above stated, and that not often; while, on the other hand, I have observed much distress to accompany the ptyalism, without any ultimate advantage; so that I cannot consider it in the light of a remedy, unless combined with a cathartic.

There is one remark I would beg to be indulged with on the publication of Mr. Pym, as he seems, in the history of the first cases of fever that occurred in his practice, to have confounded those very diseases which he wishes to be understood as possessing a distinct character, and to have designated as "Bulam fever" that which was obviously the consequence of miasmata. I allude to that which was so prevalent at Fort Edward, Martinique; the situation of which, being almost surrounded with deep moats, and to leeward of the Lemantin marshes, has always been considered unfriendly to health.

Had the epidemic of the 70th regiment been really infectious, the removal of the troops would not so speedily have destroyed its properties, as the situation was only changed for one very little more elevated, and not differing materially in temperature. Subsequent attacks of illness among troops quartered there, have led to similar measures, and with equal success.

During my residence at the Naval Hospital in that island, I have several times witnessed fever succeeding to great heats after heavy rains; and that while the transports in the bay were losing many men, and fever was commonly in the fort, the Carenage, more screened from the influence of the marsh, was perfectly healthy, although it was at that time the chief depôt for the prisoners of war.

For farther information respecting the effects of miasmata on men quartered at Fort Edward, I beg to refer to Mr. Forbes, lately Deputy Inspector of Hospitals at that island.

The preceding pages consist chiefly of matters of fact that have come more immediately under my own observation, as I conceived the faithful relation of them preferable to the devious path of conjecture, in endeavouring to elucidate the points in discussion.

JOHN MORTIMER.

---

